# Feature selection and classification for microarray data analysis: Evolutionary methods for identifying predictive genes

**DOI:** 10.1186/1471-2105-6-148

**Published:** 2005-06-15

**Authors:** Thanyaluk Jirapech-Umpai, Stuart Aitken

**Affiliations:** 1 School of Informatics, The University of Edinburgh, Edinburgh EH8 9LE, United Kingdom

## Abstract

**Background:**

In the clinical context, samples assayed by microarray are often classified by cell line or tumour type and it is of interest to discover a set of genes that can be used as class predictors. The leukemia dataset of Golub *et al. *[1] and the NCI60 dataset of Ross *et al. *[2] present multiclass classification problems where three tumour types and nine cell lines respectively must be identified. We apply an evolutionary algorithm to identify the near-optimal set of predictive genes that classify the data. We also examine the initial gene selection step whereby the most informative genes are selected from the genes assayed.

**Results:**

In the absence of feature selection, classification accuracy on the training data is typically good, but not replicated on the testing data. Gene selection using the RankGene software [3] is shown to significantly improve performance on the testing data. Further, we show that the choice of feature selection criteria can have a significant effect on accuracy. The evolutionary algorithm is shown to perform stably across the space of possible parameter settings – indicating the robustness of the approach. We assess performance using a low variance estimation technique, and present an analysis of the genes most often selected as predictors.

**Conclusion:**

The computational methods we have developed perform robustly and accurately, and yield results in accord with clinical knowledge: A Z-score analysis of the genes most frequently selected identifies genes known to discriminate AML and Pre-T ALL leukemia. This study also confirms that significantly different sets of genes are found to be most discriminatory as the sample classes are refined.

## Background

Microarray technology has provided biologists with the ability to measure the expression levels of thousands of genes in a single experiment. The vast amount of raw gene expression data leads to statistical and analytical challenges including the classification of the dataset into correct classes. The goal of classification is to identify the differentially expressed genes that may be used to predict class membership for new samples. The central diffculties in microarray classification are the availability of a very small number of samples in comparison with the number of genes in the sample, and the experimental variation in measured gene expression levels. While very effective methods for binary classification (i.e. classification into two classes) are known, these methods do not necessarily perform as well in the multi-class case [4]. This paper addresses the multi-class classification of microarray data, and the evaluation issues that arise in determining the validity of the performance measures.

The classification of gene expression data samples involves feature selection and classifier design. Feature selection identifies the subset of differentially-expressed genes that are potentially relevant for distinguishing the classes of samples. The aim is to reduce the initial gene pool from 7,000–10,000 to 100–200. Several gene selection methods based on statistical analysis have been developed to select these predictive genes, they include t-statistics, information gain, twoing rule, the ratio of between-groups to within-groups sum of squares (BSS/WSS) and principal component analysis [4,5]. In this study we explore the alternative methods provided by the RankGene software [3] for the initial feature selection task.

Both supervised and unsupervised classifiers have been used to build classification models from microarray data. This study addresses the supervised classification task where data samples belong to a known class. Many classifiers have been used for this task such as Fisher Linear Discrimination Analysis, Maximum Likelihood Discriminant Rules, Classification Tree, Support Vector Machine (SVM), K Nearest Neighbour (KNN), and aggregated classifiers [4]. In this study we adopt the KNN classifier. KNN classification is based on a distance function such as the Euclidean distance or Pearson's correlation that is computed for pairs of samples in N-dimensional space. Each sample is classified according to the class memberships of its k nearest neighbours, as determined by the distance function. KNN has the advantages of simple calculation and the ability to perform well on data sets that are not linearly separable, often giving better performance than more complex methods in many applications (e.g. [4]). The aim of this study is to evaluate an evolutionary algorithm for multiclass classification of microarray samples by assessing its classification accuracy on microarray samples. We also investigate the feature selection step that is a necessary precursor to classification. These objectives require an appropriate evaluation method to determine the final figures for accuracy. Once the appropriate parameters for the evolutionary algorithm are determined, its performance is evaluated again using the .632 bootstrap estimation method to obtain a low-variance measure. Two published microarray datasets are used to test the performance of the algorithms, namely, the leukemia and NCI60 datasets. The contributions of this paper are: a comprehensive evaluation of an evolutionary classifier; an investigation of feature selection in learning classifiers; an analysis of frequently selected genes, and a comparison of gene rankings across several previous studies of the leukemia data.

## Systems and methodology

### Evolutionary algorithm

Evolutionary algorithms have been applied to microarray classification in order to search for the optimal or near-optimal set of predictive genes on complex and large spaces of possible gene sets. Evolutionary algorithms are stochastic search and optimisation techniques that have been developed over the last 30 years. These algorithms are based on the same principles of evolution found in the biological world involving natural selection, and survival of the fittest. Evolutionary algorithms differ from other traditional optimisation techniques in that they involve a parallel search through a population of solutions.

The evolutionary algorithm we employ maintains a population of predictors whose effectiveness as a classifier can be determined by using them as features in a KNN classifier. The size of the population and the number of features in a predictor are parameters that we shall explore experimentally in the following section.

**Figure 1:**
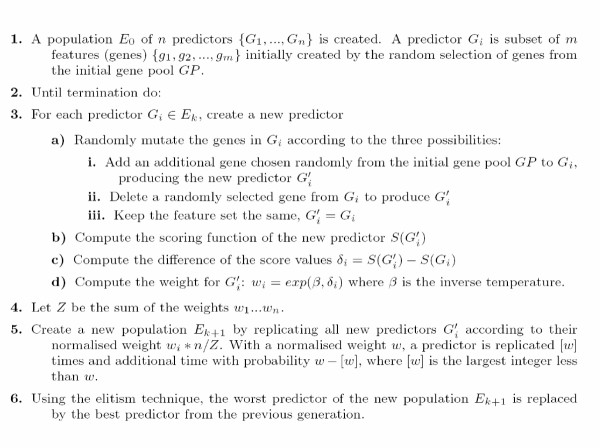
Evolutionary algorithm for multiclass classification.

We assume that an initial gene pool of informative genes has been identified (*GP*). The initial predictors in the population are randomly constructed from the initial gene pool as indicated in Figure 1. A predictor contains between 10 and 50 genes and so defines a subset of the features (genes) that are identified in the initial feature selection step.

Each predictor is scored according to its ability to classify the training data based on a leave-one-out cross validation (LOOCV) analysis. The scoring function *S *in steps 3b and 3c in Figure 1 is the sum of the training samples that are correctly classified in the LOOCV. That is, one of the samples is left out to be a pseudo test data, the classifier is built based on all but the left out sample, and the score incremented if the sample is correctly classified. The score is incremented by an additional amount proportional to the minimum separation of the clusters. The replication of a particular predictor depends on how the mutation effects the scoring function. The selection process uses a statistical replication algorithm. The predictors which have a higher score after mutation survive in the next generation. The termination condition is met when the all predictors give similar score over a specific number of generations. The most successful predictor is the one giving the fewest errors on the training samples. To measure the performance of the algorithm, the best predictor is evaluated again using test samples.

The parameters of the evolutionary algorithm are fixed as follows: The probability that a predictor will be mutated is set to 0.7. The probability of adding a new gene to the predictor and the probability to delete a gene from any random position in predictor are set to 0.5. This makes adding a gene and deleting a gene equally probable. The KNN classifier uses the Euclidean distance measure. When surveying the performance for the population size and feature size parameters we report the average of the accuracy rate over 10 trials.

The selection and evolution process is a modification of the statistical replication algorithm described in Deutsch [6]. The termination criteria is defined using both the maximum number of generations and the criteria of no improvement of maximum fitness value of the population. The predictor with highest fitness will be the one that contains the best subset of genes for the classification task.

### Genetic algorithm

In order to confirm the conclusions drawn from the experimental analysis, we repeat the classification task using a different search algorithm – a standard genetic algorithm (GA) – in combination with a k-nearest neighbour classifier. The GA encodes a weight for each feature in a chromosome, and maintains a population of chromosomes which are combined using crossover, and modified by mutation operators in a standard manner [7]. The GA explores the space of weights for each feature using a 1 bit representation, where the feature is assigned a weight of 0 or 1. In this configuration, the GA+KNN classifier performs feature selection and hence is directly comparable to the evolutionary algorithm. The GA was configured to have 20 chromosomes and was run for 100 generations in each trial.

### Methodology

To investigate the initial feature selection step, and its effect on classifier learning, we apply six of the feature selection methods supported in the RankGene [3] software. These techniques have been widely used either in machine learning (e.g. information gain and Gini index) or in statistical learning theory (e.g. twoing rule, max minority, sum of variances). They quantify the effectiveness of a feature by evaluating the strength of class predictability when the prediction is made by splitting the full range of expression of a given gene into two regions, the high region and the low region. The split point is chosen to optimise the corresponding measure. Feature selection is performed using only the samples in the training data set.

The evolutionary algorithm that we shall evaluate learns the optimal subset of features that classify the data, based on the initial set of selected features. In the trials reported here, the evolutionary algorithm will stop when the score of all predictors in the population give a standard deviation of less than 0.01 for ten consecutive generations, or the evolutionary algorithm reaches the maximum number of generations (200).

Many evaluation methods has been investigated for small-sample error estimation. Typically, a microarray experiment provides a dataset of small size, and as a result the most commonly used method for error estimation is leave-one-out cross validation (LOOCV). The LOOCV error rate estimator is a straightforward technique and gives an almost unbiased estimator.

A comparison of various error estimation methods is presented in [8], these include the resubstitution estimator, k-fold cross-validation, leave-one-out estimation, and bootstrap methodology. These methods were tested with many classifiers: linear discriminant analysis, 3-nearest-neighbour, and decision trees. For over all performance, the .632 bootstrap proved to be the best estimator in their simulations, but the drawback of this method is the computational cost in comparison to LOOCV. As LOOCV is almost unbiased and is fast, it is acceptable to use it for parameter analysis.

We investigate the performance of the feature selection methods and evolutionary algorithm as follows:

• The evolutionary classifier is tested on the leukemia dataset without the use of any feature selection method.

• The RankGene methods are employed, and performance compared against the baseline and against each other for the leukemia data.

• The GA-based classifier is evaluated on the leukemia data to validate these results.

• The evolutionary classifier is tested on the leukemia and NCI60 datasets.

• A ranking of frequently selected genes by Z-score is obtained, and the performance of the top ranked genes as a classifier is measured.

• The .632 bootstrap error estimator is applied using the optimal parameters for all datasets.

## Results

**Figure 2:**
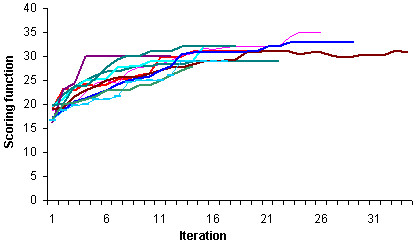
The average score in each iteration for several trails of the evolutionary algorithm on the leukemia dataset.

We begin by demonstrating that the performance of the population of predictors improves on each iteration of the evolutionary algorithm. Figure 2 shows the average scores in each iteration from several trials. The graph shows that the average score increases more rapidly over the first few generations in comparison with the final generations. The evolutionary algorithm typically converges and terminates in less than 50 generations. The runs terminate on different iterations depending upon when the termination condition is met.

In the baseline test, the evolutionary algorithm is run on the entire set of genes (without applying any feature selection method) in order to obtain a baseline measure of performance.

The baseline system is evaluated on the 7,070 genes of the leukemia dataset. The initial predictors in population are built by randomly selecting 10 genes to be an initial feature of the predictors. This means the evolutionary algorithm has to search for 10 optimal predictive genes set from the  possible subsets. Performance of the predictors is evaluated using KNN classifier to determine the LOOCV on training samples. After the best predictor is found in each generation, it will be tested again on test samples to give the performance based on an out-of-sample estimation. The KNN classifier classifies each sample in the test data using a database of all training samples.

Table 1 reports the maximum and the average accuracy of the baseline system on 38 training samples and 34 test samples. The results show that the evolutionary algorithm gives predictors with perfect classification on the training samples but those predictors do not classify the test data well. The average accuracy on test data is 68% at best while the average accuracy on training data is up to 98%. Table 1 indicates that population size may be a more important factor than feature size for the baseline system.

**Table 1:** The accuracy of the baseline system built by randomly selecting genes from 7,070 genes in the leukemia dataset.

Population size	Feature size	Training data [%]		Test data [%]	
		
		Max	Average	Max	Average
10	30	89.47	85.79	79.41	64.41
	50	89.47	84.21	76.47	63.53
30	30	94.74	98.42	85.29	68.82
	50	97.37	96.05	73.53	67.35
50	30	100.00	98.42	85.29	70.29
	50	100.00	98.42	85.29	72.64

### Building an informative initial gene pool by the RankGene software

We determine the best choice for the number of initial features, i.e. the initial gene pool, to be 100 by experiment (by exploring the performance of the classifier using the information gain ranking method for feature selection). Based on this result, the performance of the following six ranking methods for a range of population and feature sizes is investigated: R1. Information gain; R2. Twoing rule; R3. Gini index; R4. Sum minority; R5. Max minority; R6. Sum of variances. The details of each method can be found at .

### Leukemia dataset

The parameters of the evolutionary classifier are evaluated on the set of 38 training samples and the 34 test samples of the leukemia data – as described in the original work. We explore: population size {10, 30, 50} feature size {30, 50} and initial gene pool 100.

The classification results are summarised in table 2. When a predictor with 100% accuracy is learned in one or more of the test runs we indicate this by (*). Average accuracy over ten trials lies in the range 92–98%. Using feature selection, the accuracy on the test data is more than 19% greater than that of the baseline system. For a given feature selection method, the prediction accuracy on the test samples varies up to 3% across the range of parameter settings surveyed. The choice of feature selection method contributes up to 5% for a given set of algorithm parameters. Information gain consistently gives the best performance on the leukemia dataset.

**Table 2:** The average accuracy using out-of-sample prediction on the 34 leukemia test samples. The symbol (*) means that there is some perfect predictors found by the algorithm. The highest accuracy is written in bold.

Population size	Feature size	The accuracy of different rank methods on the Test data (out-of-sample) [%]					
		
		R1	R2	R3	R4	R5	R6
10	30	97.35*	95.29	93.82	92.94*	93.53	94.12
	50	98.24*	95.59	94.71*	93.82	93.53	95.00
30	30	96.74*	92.65	94.41	95.00*	94.71	93.82
	50	97.06*	95.00	95.00	93.82	95.30	93.82
50	30	97.35*	93.82	94.71*	92.06*	94.12*	93.82*
	50	96.17	93.82	92.65	92.35	94.12	94.71

Abbreviations: R1. Information gain; R2. Twoing rule; R3. Gini index; R4. Sum minority; R5. Max minority; R6. Sum of variances.

The results of the confirmation study are shown in table 3, where feature selection improves accuracy by at least 27%: The accuracy on the training data in the baseline system (without feature selection) is 85.26% which is significantly greater than the test accuracy of 67.06%. A variation of 4.4% is observed in average accuracy across the RankGene methods. The best RankGene method is information gain. In comparison with feature selection by information gain, all other methods have a significantly lower accuracy on the testing data than information gain, while there are no differences in performance on the training data across the other RankGene methods.

**Table 3:** The average accuracy of the GA/KNN classifier using out-of-sample prediction on the 34 leukemia test samples.

The accuracy of different rank methods on the Test data (out-of-sample) [%]						
Baseline	R1	R2	R3	R4	R5	R6

67.06	98.24	95.29	93.82	95.00	94.71	95.00*

### NCI60 dataset

The aim of this investigation is to find the best parameters and ranking method for the evolutionary classifier when applied to the NCI60 data. The same range of parameters is surveyed as for the leukemia data, and again the performance of six feature selection methods is evaluated.

Due to the very small sample size of the NCI60 dataset, it is not possible to divide the data into training and testing sets. Thus, the accuracy of predictors in table 4 is given by using the LOOCV error rate estimation on the whole dataset. To get a more reliable performance of the evolutionary algorithm on the NCI60 dataset, the .632 bootstrap estimator will be used.

**Table 4:** The average accuracy on 5 sets of parameters and six ranking methods on the NCI60 data. No perfect predictors are found by the algorithm. The highest accuracy is written in bold.

Population size	Feature size	The accuracy of different rank methods on all dataset (LOOCV) [%]					
		
		R1	R2	R3	R4	R5	R6
10	30	66.72	63.77	60.00	54.43	62.78	69.34
	50	67.86	62.78	61.63	52.62	62.62	65.90
30	30	**76.23**	72.29	72.02	65.90	74.26	75.41
	50	73.44	72.46	71.15	63.11	73.44	73.93
50	50	75.08	72.29	71.96	71.97	73.77	74.16

The best classification score on the NCI60 data is 76.23%, and was obtained using information gain, with a population and feature size of 30. No predictor learned in any run of the system was able to classify all data 100% correctly.

### Discrimination method

The frequency of selection of the genes that are members of the best predictor across 100 independent trials is assessed in order to determine the reproducibility of the results. If a gene is consistently preferentially chosen as a member of a predictive set it would suggest that the gene selection operation is reproducible – despite the random initialisation.

Z-score analysis is one means to determine the significance of the observed frequency of an event against that which might have occurred by chance. This calculation normalises the frequency with which each of the initial genes was selected in all predictors that classify the training and test data perfectly [9]. The Z score can be calculated using equation (1).



Where *S*_*i *_denotes the number of times genes *i *was selected, *E*(*S*_*i*_) is the expected number of times for gene *i *being selected, and *σ *denotes the square root of the variance. The calculation of *E*(*S*_*i*_) is as follows: let *A *number of perfect predictors found in the experiment, *P*_*i *_= (number of genes) / (number of genes in the initial gene pool). Then, *E*(*S*_*i*_) = *P*_i _* *A*.

**Figure 3:**
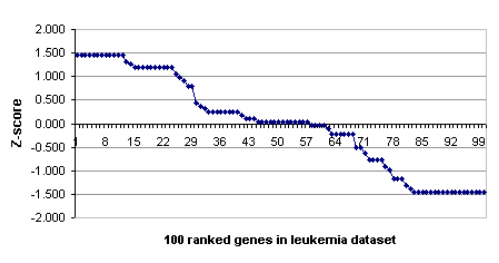
A plot of Z-scores for 100 ranked genes on the leukemia dataset.

The evolutionary classifier was run 100 times on the leukemia dataset using the best set of parameters: 100 initial genes constructed by the information gain and feature size = 50. There are 43 predictors (ignoring the duplication) that classify all training and test data correctly. Figure 3 shows a plot of Z-score applied to the top ranked genes that are most frequently selected. The top 24 genes have a similar Z-score and the top 55 have a positive Z-score. In this case, it seems reasonable to choose the 55 top-ranked gene as the most discriminative genes.

To confirm the predictive power of the 55 top-ranked genes we constructed a classifier using these genes as features. As before, we measure the accuracy on the test set using the training set as a case-base. An accuracy of 100% is obtained.

Additional file: 1 shows the 55 top-ranked genes, their frequency of occurrence in the 43 best predictors and the Z-score value. This set of genes will be used in the further evaluation using .632 bootstrap estimator.

**Figure 4:**
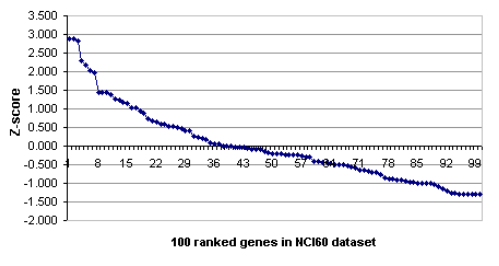
A plot of Z-scores for 100 ranked genes on the NCI60 dataset.

The Z-score analysis was repeated on the NCI60 data and these results are shownin Figure 4. It can be seen that the Z-score of the 8th ranked gene is half that of the top ranked gene and that the top 40 genes have a positive Z-score. Additional File 2 lists the top 40 genes. A classifier constructed using these genes has an accuracy of 73.8% by LOOCV, and 68.2% by a bootstrap estimate.

### .632 bootstrap estimates

The .632 bootstrap method involves sampling the original dataset (with replacement) to obtain a new resampled set on which the classification error *δ *is measured [8]. The accuracy *ε *on the samples omitted from the resampled set is also determined. The bootstrap estimator *a*_*b*632 _for a dataset of *n *samples requires *n *resampled datasets to be constructed and the classification error calculated according to equation (2). The final bootstrap estimate is the average value of *a*_*b*632 _over *b *iterations of the procedure (we take *b *= 200).



On the leukemia data, the .632 bootstrap estimate for the accuracy of the evolutionary algorithm with population size 10 and feature size 50 is 96.40%. The .632 estimate for a classifier based on a fixed set of 55 top-ranked genes is 96.85%.

On the NCI60 data, the .632 bootstrap estimate for the accuracy of the evolutionary algorithm with population size 30 and feature size 30, is 59.19%. The .632 estimate for a classifier based on 40 top-ranked genes is 68.18%.

## Discussion

On the data sets studied, we find that the evolutionary algorithm performs robustly over the space of parameters surveyed. Optimal values for population size and feature size are obtained, but the approach is not overly sensitive to these choices. This is a promising result as stochastic algorithms and techniques such as neural nets can be sensitive to parameter settings [10].

Despite showing approximately 97% accuracy on the leukemia data, the evolutionary algorithm does not perform as well on the NCI60 dataset. The .632 bootstrap estimate of 59% for the NCI60 data is significantly less than the 76% obtained by LOOCV. If we accept that the bootstrap method is appropriate, then we must conclude that the LOOCV estimates typically reported may be considerable overestimates. The LOOCV estimate we obtain is greater than the averages for the NCI60 data reported in [4,5], but less than the best error rate reported in [11]. The bootstrap estimate is comparable with the results of [5].

### Gene selection

The value of the RankGene methods for feature selection is shown by the improvement in accuracy over the baseline results. Without feature selection, the evolutionary algorithm can discover predictors that give 100% accuracy on the training set, but perform poorly on the test set. A similar finding is obtained using a GA-based search with a KNN classifier. This indicates that the classes can be distinguished by any of a large set genes that are indicative of a category, but that these genes are not necessarily informative in the sense that they are activated in a comparable way across both the training and the testing sets. By allowing uninformative genes into the feature set we observe a degree of overfitting.

The correct choice of RankGene method can improve classification by 5% for a given population size and feature size on the leukemia data (when using the evolutionary algorithm). The confirmation study reproduced this finding also. In comparison, the selection of population and feature sizes can influence performance by up to 3% (for a given RankGene method). Thus, feature selection has a significant influence on the classifier learning task.

### The optimal predictor

Several researchers have tried to find the optimal predictive gene set. It has been suggested that for the leukemia dataset the number of predictive genes included in a predictor should be less than 50 [12]. This conclusion was based on a Z-score analysis. Deutsch [6] found an average of 9 predictors was sufficient to classify the leukemia data perfectly, with some runs reducing the number to 2. We find 10 genes are not sufficient, and that that up to 55 genes are required, but have not attempted to identify a lower bound.

### Gene ranking

For comparison, the rankings of selected genes found to be significant in previous analyses [13,14] of the leukemia data are listed in Table 5 along with those ranked highly by our methods. The ranking of Ben-Dor [13] is of genes that discriminate between AML and ALL, while Thomas [14] ranks the 25 genes more highly differentially expressed genes in AML than in ALL, and provides a similar ranking for the genes differentially expressed in ALL.

**Table 5:** Top ranking genes by Z-score (top 24 genes, this paper), by TNoM score (top 30 genes [13]^1^), and by differential expression in AML or in ALL (top 25 genes, [14]^2^). As the Z-score can give genes equal scores, a rank of 1 can be assigned to several genes. In our study 12 genes are ranked 1, in that of [13] 3 are ranked 1. The lowest place in a total ordering of the genes is indicated by figure in parenthesis, e.g. (≤12). Genes ranked in column 5 cannot appear in column 6, and vice versa, as indicated by (-). Otherwise, a blank entry indicates a ranking outside of the top 24, 25 or 30 genes respectively.

Gene	Description	Rank by Z-score	Rank by TNoM^1^	Rank in AML^2^	Rank in ALL^2^
M23197	Myeloid cell surface antigen CD33	1 (≤12)	1 (≤3)	22 (≤22)	-
X04145	T-cell surface glycoprotein CD3	1 (≤12)			
M31211	Myosin light chain	1 (≤12)	6 (≤20)	-	4 (≤4)
M31303	Leukemia-associated phosphoprotein	2 (≤13)	7 (≤32)	-	11 (≤13)
U50136	Leukotriene C4	3 (≤24)	7 (≤32)	3 (≤3)	-
M28170	B-lymphocyte surface antigen CD19	3 (≤24)			
J04132	T-cell surface glycoprotein CD3	3 (≤24)			
X95735	Zyxin		1 (≤3)	6 (≤6)	-
M55150	Fumarylacetoacetate		6 (≤20)	1 (≤1)	-
X59417	Proteasome iota chain		6 (≤20)	-	3 (≤3)
U22376	C-myb			-	1 (≤1)

Table 5 indicates that the genes that rank highly by differential expression lie outside of the top 15 in the rankings that are derived to support classification, and some rank outside of the top 30 (as indicated by a blank entry in the table). For example, M23197, which is the human differentiation antigen CD33 and a known indicator of myeloid lineage [15], is ranked first the classification-based scores but is 22nd in terms of differential expression.

Only four of the top 24 genes we identify are found in the 50 genes listed as having highest relative differential expression for AML/ALL respectively. In contrast, 14 of the top 30 genes of Ben-Dor [13] are in this list (only a selection are tabulated here). This difference can be accounted for by the fact that we address the 3-class problem while Ben-Dor solve the 2-class problem. Thus it appears that the differential expression of genes can be exploited in classifying samples but, even in the 2-class case will, not lead to optimal classification as it ignores the relationships between the expression levels in a predictive set of genes.

Five of the genes we rank in the top 3 (range 1–24 in terms of a total ordering of genes) also lie in the equivalent range (rank 1–6, or range 1–20 in a total ordering) in [13]. Further, the human CD19 antigen gene M28170 and T-cell surface glycoproteins XO4145 and J04132 which are ranked in the top 24 in our study do not appear in the top 50 genes in Ben-Dor [13] or in Thomas [14]. CD19 is known to indicate B-lineage and CD3 to indicate T-lineage [15] This study confirms that significantly different sets of genes are found to be most discriminatory as the sample classes are refined.

## Conclusion

The evolutionary methods we have developed for microarray data classification perform robustly and accurately on the data sets examined. The results are in accord with clinical knowledge as demonstrated by a Z-score analysis of the genes most frequently selected for inclusion in a classifier: Genes known to discriminate between AML and Pre-T ALL leukemia are identified. This study also concludes that there are notable dependencies between the way in which the classification problem is formulated and the resulting rankings of discriminatory genes.

## Methods

The 3-class leukemia dataset [1] and the 9-class tumour NCI60 dataset [2] are used in this study. The leukemia dataset is available at . In this dataset, gene expression levels were measured using Affymetrix high-density oligonucleotide arrays containing 6,817 genes. The dataset consists of 47 samples of acute lymphoblastic leukemia(ALL) and 25 samples of acute myeloblastic leukemia(AML). Originally, the dataset was built and analysed for binary classification: ALL and AML. However, it can be separated into three or four classes by using subtypes of ALL. To perform a multiclass classification task, 72 samples in the dataset are divided into three classes: ALL B-CELL(38), ALL T-CELL(9), and AML(25). The training set is composed of 27 samples of ALL and 11 samples of AML, and the test set is composed of 20 ALL and 14 AML samples. Golub *et al. *[1] have normalised the dataset by re-scaling intensity values to make the overall intensities for each chip equivalent and also fitted the data with a linear regression model. From 7,129 genes, the baseline genes were cut off before further analysis. The number of genes that are used in the multiclass classification task is 7,070.

The NCI60 dataset is available at . This dataset contains the gene expression profiles of 64 cancer cell lines measured by cDNA microarrays and is provided without normalisation. The gene expression data value is the relative intensity level of the mRNA samples and their references. The single unknown cell line and two prostate cell lines were excluded from analysis due to their small number. Nine classes of samples (61 cell lines) are identified: breast (7), central nervous system (5), colon (7), leukemia (6), melanoma (8), non-small-cell-lung-carcinoma or NSCLC (9), ovarian (6), renal (9) and reproductive (4). Excluding negative and missing values, the number of genes considered is reduced from 9,703 to 7,375 genes.

Due to the noisy nature of the datasets resulting from microarray experiments, preprocessing is an important step. The NCI60 dataset has to be normalised to decrease the variation before feature selection or classification. Global normalisation is used to eliminate systematic variance. For NCI60 dataset, the analysis of relative gene expression level is done by using the log-ratios between a certain gene (labelled in red or Cy5) and a reference gene (labelled in green or Cy3) before the data is normalised. The normalised values *M *are given by (3) where the constant *c *is estimated from the mean for the log-ratio *log*_2 _(*R*/*G*).

*M *= *log*_2 _(*R*/*G*) - *c *    (3)

## Authors' contributions

TJU designed and implemented the evolutionary classifier, and carried out the experiments. SA worked with TJU on the design of the study and helped to draft the manuscript. Both authors read and approved the final manuscript.

## Supplementary Material

Additional file 1 The 55 top-ranked leukemia genes ordered by the frequency that the gene is selected. Click here for file

Additional file 2 The 40 top-ranked NCI60 genes ordered by the frequency that the gene is selected. Click here for file
