# Supplementary material for: Feature selection and classification for microarray data analysis: Evolutionary methods for identifying predictive genes
Source: BMC Bioinformatics. 2005 Jun 15;6:148. doi: 10.1186/1471-2105-6-148 (PMC1181625; doi:10.1186/1471-2105-6-148)
Supplement: Additional file 1 — The 55 top-ranked leukemia genes ordered by the frequency that the gene is selected. [file 1471-2105-6-148-s1.pdf]

Additional File 1

The 55 top-ranked leukemia genes ordered by the frequency that the gene is selected.

Gene Index	Gene Access Number	Frequency	z-score value
1775	M23197_at	43	1.458
3996	X04145_at	43	1.458
4023	X05908_at	43	1.458
6451	U23852_s_at	43	1.458
6222	M31211_s_at	43	1.458
3222	U48251_at	43	1.458
4523	X76648_at	43	1.458
1720	M19507_at	43	1.458
4048	X07743_at	43	1.458
6108	M12959_s_at	43	1.458
2936	U28833_at	43	1.458
4585	X80230_at	43	1.458
1869	M31303_rna1_at	41	1.323
4314	X62320_at	40	1.255
701	D88422_at	39	1.187
6177	M23323_s_at	39	1.187
2302	M92934_at	39	1.187
4283	X59871_at	39	1.187
3261	U50136_rna1_at	39	1.187
6915	M28170_at	39	1.187
1770	M22960_at	39	1.187
1047	J04132_at	39	1.187
6109	M13560_s_at	39	1.187
3983	X03100_cds2_at	39	1.187
1951	M38690_at	37	1.052
2774	U16954_at	36	0.985
2289	M91432_at	35	0.917
1571	L47738_at	33	0.782
5897	J03077_s_at	33	0.782
6126	X64072_s_at	28	0.444
2655	U09578_at	27	0.376
863	HG3494-HT3688_at	26	0.309
1626	M11722_at	25	0.241
6317	M83652_s_at	25	0.241
3991	X03934_at	25	0.241
1019	J03473_at	25	0.241
6514	U49020_cds2_s_at	25	0.241
5276	M21535_at	25	0.241
1098	K01396_at	25	0.241
2363	M98045_at	25	0.241
2062	M63138_at	24	0.174
3049	U36922_at	23	0.106
2343	M96326_rna1_at	23	0.106
6438	X74301_s_at	23	0.106
5629	L33930_s_at	22	0.039
4307	X61587_at	22	0.039
1100	K01911_at	22	0.039
4318	X62654_rna1_at	22	0.039
6666	U46006_s_at	22	0.039
5241	L08895_at	22	0.039
4137	X17042_at	22	0.039
5407	X58072_at	22	0.039
1686	M16038_at	22	0.039
6480	X85116_rna1_s_at	22	0.039
6319	M83667_rna1_s_at	22	0.039
