# Supplementary material for: Feature selection and classification for microarray data analysis: Evolutionary methods for identifying predictive genes
Source: BMC Bioinformatics. 2005 Jun 15;6:148. doi: 10.1186/1471-2105-6-148 (PMC1181625; doi:10.1186/1471-2105-6-148)
Supplement: Additional file 2 — The 40 top-ranked NCI60 genes ordered by the frequency that the gene is selected. [file 1471-2105-6-148-s2.pdf]

Additional File 2

The 40 top-ranked NCI60 genes ordered by the frequency that the gene is selected.

Gene Index	Gene Access Number	Frequency	z-score value
3682	6291	1097	2.873
4491	7333	1097	2.873
455	1029	1084	2.824
836	1892	947	2.303
3957	6608	916	2.185
3959	6610	874	2.026
2362	4629	862	1.980
4378	7171	724	1.456
1340	3070	719	1.437
1976	4073	718	1.433
3105	5536	707	1.391
1781	3763	670	1.250
1889	3927	663	1.224
4368	7156	654	1.189
5375	8454	646	1.159
906	2059	613	1.034
6283	9588	609	1.018
4413	7226	585	0.927
726	1676	574	0.885
5752	8937	533	0.730
5728	8911	518	0.673
4241	6990	508	0.635
4741	7676	498	0.597
5605	8754	493	0.578
1619	3529	481	0.532
6218	9521	477	0.517
2664	4995	475	0.509
5826	9029	463	0.463
2889	5272	453	0.425
2612	4933	446	0.399
5666	8837	409	0.258
5749	8934	400	0.224
6271	9576	392	0.194
4480	7321	386	0.171
2417	4700	363	0.083
5306	8363	358	0.064
2907	5296	357	0.061
5810	9009	344	0.011
5472	8572	344	0.011
5378	8457	343	0.007
